# Brigadier General George Henry Torney, M. D.

**Published:** 1914-02

**Authors:** 


Brigadier General George Henry Torney, M. D., University of Virginia 1870, Surgeon General, U. S. A., died in Washington, December 27, 1913, aged 63. The cause of death was broncho-pneumonia.

